# A Hybrid Soft Sensor Model for Measuring the Oxygen Content in Boiler Flue Gas

**DOI:** 10.3390/s24072340

**Published:** 2024-04-07

**Authors:** Yonggang Wang, Zhida Li, Nannan Zhang

**Affiliations:** College of Information and Electronic Engineering, Shenyang Agricultural University, Shenyang 110866, China; wygvern@syau.edu.cn (Y.W.); lizhida@stu.syau.edu.cn (Z.L.)

**Keywords:** soft sensor model, oxygen content in flue gas, long short-term memory, whale optimization algorithm

## Abstract

As an indispensable component of coal-fired power plants, boilers play a crucial role in converting water into high-pressure steam. The oxygen content in the flue gas is a crucial indicator, which indicates the state of combustion within the boiler. The oxygen content not only affects the thermal efficiency of the boiler and the energy utilization of the generator unit, but also has adverse impacts on the environment. Therefore, accurate measurement of the flue gas’s oxygen content is of paramount importance in enhancing the energy utilization efficiency of coal-fired power plants and reducing the emissions of waste gas and pollutants. This study proposes a prediction model for the oxygen content in the flue gas that combines the whale optimization algorithm (WOA) and long short-term memory (LSTM) networks. Among them, the whale optimization algorithm (WOA) was used to optimize the learning rate, the number of hidden layers, and the regularization coefficients of the long short-term memory (LSTM). The data used in this study were obtained from a 350 MW power generation unit in a coal-fired power plant to validate the practicality and effectiveness of the proposed hybrid model. The simulation results demonstrated that the whale optimization algorithm–long short-term memory (WOA-LSTM) model achieved an *MAE* of 0.16493, an *RMSE* of 0.12712, an *MAPE* of 2.2254%, and an $R^{2}$ value of 0.98664. The whale optimization algorithm–long short-term memory (WOA-LSTM) model demonstrated enhancements in accuracy compared with the least squares support vector machine (LSSVM), long short-term memory (LSTM), particle swarm optimization–least squares support vector machine (PSO-LSSVM), and particle swarm optimization–long short-term memory (PSO-LSTM), with improvements of 4.93%, 4.03%, 1.35%, and 0.49%, respectively. These results indicated that the proposed soft sensor model exhibited more accurate performance, which can meet practical requirements of coal-fired power plants.

## 1. Introduction

With the progression of technology and growing emphasis on environmental preservation, it becomes exceptionally vital to optimize coal’s utilization efficiency and enhance the productivity of coal-fired power plants [1]. This is essential to meet the demands of energy conservation, emission reductions, low-carbon practices, and sustainable development. Boilers serve as the fundamental equipment in coal-fired power stations, which directly determine the economic benefits and the efficiency of generation for the whole generator unit. A critical indicator of the state of combustion within the boiler is the oxygen content in the flue gas. The oxygen content not only affects the thermal efficiency of the boiler and the energy utilization of the generator unit, but also has adverse impacts on the environment. Therefore, accurately measuring the oxygen content of flue gas is important in enhancing the energy utilization efficiency of coal-fired power plants and reducing the emissions of waste gas and pollutants [2].

In current industrial processes, there are various approaches for measuring the oxygen content in flue gas, which can be primarily categorized as soft measurement techniques [3,4] and direct measurement [5]. The direct measurement method uses instruments such as zirconia oxygen analyzers or thermomagnetic oxygen analyzers to measure the oxygen content. However, these conventional oxygen sensors have certain limitations, including time delay, declining measurement accuracy due to the sensor aging, steep hardware replacement costs, and a constrained equipment lifespan. Consequently, conventional oxygen sensors fail to ensure continuous real-time monitoring, resulting in the inability to achieve optimal combustion conditions [6,7,8]. 

To address the problem of conventional oxygen sensors, the soft measurement method is proposed, which utilizes virtual sensing technology to estimate crucial variables based on other accessible process variables. By integrating data processing, mathematical modeling, and software technologies, the soft measurement method presents a feasible and cost-effective alternative, mitigating the constraints associated with traditional measurement techniques. The soft sensor methods in industrial processes based on data-driven methods include mixture Gaussian process (GP) [9], principal component analysis (PCA) [10], support vector regression (SVR) [11], artificial neural networks (ANNs) [12], and so on. Through utilization of PCA for extracting flame data and GP for constructing a stochastic model of the combustion process, a rapid estimation of the oxygen concentration in the flue gas was achieved [13]. A soft measurement technique for flue gas oxygen content was developed based on the SVR method, exhibiting superior accuracy and generalization capability compared with other approaches [14]. Considering a 600 MW coal-fired power plant unit with a boiler combustion system, a soft-sensing model for oxygen content based on the backpropagation neural network (BPNN) was presented [15]. Furthermore, by integrating the BPNN and the genetic algorithm (GA), a soft measurement approach for measuring the oxygen content in a power plant’s flue gas was proposed [16]. A method combining extreme gradient boosting (XGBoost) and ANN was proposed to predict the oxygen content in boilers [17]. By implementing the aforementioned method, satisfactory experimental results could be obtained via effective measurement of the oxygen content in the flue gas. However, traditional machine learning techniques are susceptible to interference and anomalies, which can disturb the extraction of the distinguishing characteristics and the proper fitting of the models, thus diminishing the models’ efficacy [18]. Moreover, in the case of temporal data, conventional machine learning approaches may fail to fully exploit the spatiotemporal of the data, resulting in poor predictive abilities.

With the development of deep learning technology in recent years, deep neural networks have emerged as a trending topic of discussion. Prominent among the widely used deep learning algorithms are deep belief networks (DBN) [19], convolutional neural networks (CNN) [20], stacked autoencoders (SAE) [21,22], and long short-term memory (LSTM) [23]. A hybrid prediction model based on DBN has been proposed to predict the oxygen content in boiler flue gas. This model improved the accuracy of predictions of the oxygen content in boiler flue gas by utilizing control variables in DBN and combining state variables with the least squares support vector machine (LSSVM) [24]. A time series convolutional neural network (TS-CNN) model using historical values of the oxygen content in boiler flue gas as the model’s input was proposed to address the problem of online prediction of the oxygen content in boiler flue gas [25]. A data-driven soft sensor modeling method based on a combination of a stacked autoencoder (SAE) and support vector regression (SVR) was proposed, analyzing the deformation of the rotor of the boiler’s air preheater in thermal power plants [26]. A weighted principal component analysis (WPCA) algorithm combining particle swarm optimization (PSO) with LSTM networks was proposed for predicting the oxygen content [27]. A method for constructing LSTM models using the Keras deep learning framework was proposed to predict the oxygen content of flue gas in boilers, replacing the real oxygen sensors [28]. To tackle the problem of lagging measurements of the NOx concentration at the boiler’s outlet in denitrification control, an innovative approach to real-time modeling of the NOx concentration at the boiler’s outlet was developed [29,30,31]. These methods used a high-precision online forecasting model based on LSTM to achieve accurate predictions of the NOx concentration. However, the architecture of LSTM is relatively complex and has many parameters, so it can be challenging to configure, which can result in lower prediction accuracy and potential overfitting issues. To overcome these issues, LSTM models widely incorporate optimization algorithms to obtain the optimal parameter settings.

In 2016, Mirjalili proposed the WOA to solve complex optimization problems, which is a clever metaheuristic optimization technique. The algorithm takes cues from the hunting behavior of whale populations and uses three different operators to simulate the pursuit and capture of prey by sperm whales, as well as their bubble-net hunting pattern. In reference [32], the WOA was tested using 29 mathematical optimization problems and six structural design problems. The optimization results showed the WOA had strong competitiveness, as proposed. A hybrid method combining hybrid differential evolution (DE) and the WOA was presented to accurately extract the parameters of the PV model [33]. The method of combining the WOA with the LSSVM was proposed to predict CO_2_ emissions, demonstrating high predictive accuracy [34]. An improved model based on an echo state network (ESN) and the WOA was proposed to predict changes in the level of liquid in a blast furnace’s gasifier [35]. This algorithm exhibits superior abilities in global search and rapid convergence, culminating in expedited model optimization. Compared with other optimization algorithms such as the GA and the PSO, the implementation process of the WOA seems to be relatively straightforward, diminishing algorithmic complexity.

In summary, this study presents a soft measurement methodology based on the WOA-LSTM model which utilizes historical production data for analysis, enabling rapid and accurate measurement of the oxygen content in flue gas. The main contributions of this study are as follows:

(1) The prediction of the oxygen content in flue gas typically involves multiple variables, which often have noise and redundant information present in the actual data. By using PCA, the high-dimensional dataset can be transformed into a lower-dimensional one, thereby enhancing the computational efficiency and accuracy of the soft measurement method. By utilizing Pearson’s method for selecting auxiliary variables and combining it with PCA, it is possible to transform high-dimensional datasets into low-dimensional ones. Additionally, the implementation of the 3*σ* criteria and smooth curve methods can effectively eliminate abnormal data, resulting in enhanced computational efficiency and accuracy in soft sensing.
(2) To the extent of our knowledge, there is a relatively limited amount of research on the use of the WOA-LSTM hybrid model for predicting the oxygen content in flue gases in soft sensor applications. To improve the precision of the LSTM model in predicting the oxygen content in flue gas, the study used the WOA optimizing the learning rate, hidden layer units, and regularization coefficient of the LSTM model. Compared with the LSTM, LSSVM, PSO-LSTM, and PSO-LSSVM models, the WOA-LSTM model achieved a high level of predictive accuracy for the oxygen content in the flue gas.

## 2. Analysis of the Coal-Fired Boiler System

**Figure 1:**
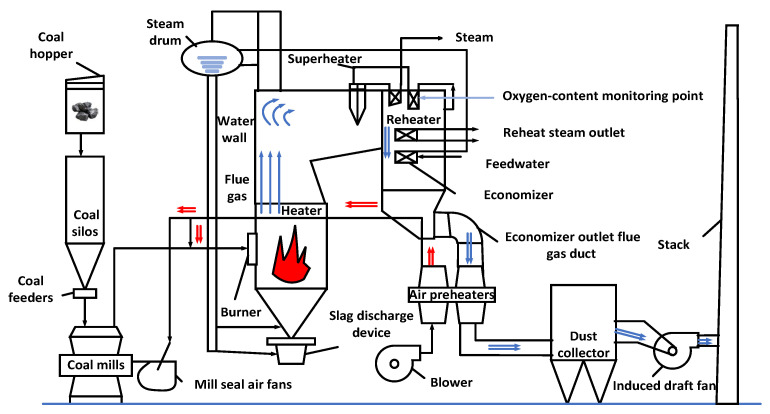
Production process of coal-fired boilers.

The basic schematic diagram of a boiler combustion system is shown in Figure 1. The operation process of this system primarily involves three parts: feeding, combustion, and emission of the flue gas [36]. Firstly, the raw coal undergoes primary crushing and removal of impurities, and then it is processed through coal hoppers, coal feeders, and coal mills to obtain qualified coal powder. Secondly, the air blower delivers cold air into the air preheater for heating. The heated air will then be divided into the primary air and secondary air. Primary air enters the coal mill, carrying the coal dust into the furnace with the aid of a blower; the secondary air provides oxygen for combustion, generating flue gas with thermal energy. The flue gas transfers heat to the water wall through radiation, causing it to produce saturated water vapor, which then rises through the rising pipes in the water wall and enters the steam drum. After being heated in the economizer, water enters the steam drum to maintain the level of liquid and transports saturated steam to the superheater. The saturated steam is heated to the temperature of superheated steam, which then enters the high-pressure cylinder of the turbine, driving the generator to produce electricity. The exhaust gas in the high-pressure cylinder of the turbine is heated by the reheater to the same temperature as the superheated steam, and then enters the intermediate-pressure cylinder and low-pressure cylinder of the turbine to undergo expansion and perform work. Unburned residual ash descends into the bottom of the furnace in the form of coarse particles and is eliminated by the slag discharge device. Finally, the residual flue gas from the reheater, economizer, and air preheater is purified of its ash particles through the dust collector, then discharged into the chimney via the induced draft fan and ultimately released into the atmosphere. The oxygen content in the flue gas can reflect the conditions of coal combustion. Under suitable oxygen conditions, fully burned coal can improve the efficiency of power generation while reducing harmful gas emissions. Therefore, it is very important to monitor the oxygen content in the flue gas in coal-fired power plants to ensure optimal combustion efficiency and environmental protection. 

Currently, the predominant method used for measuring the oxygen content in flue gas within coal-fired power plants involves the utilization of zirconia sensors to monitor this parameter. Zirconia sensors operate on the basis of measuring the difference potential of the oxygen concentration, enabling the quantification of the oxygen content [37]. These sensors have reference and measurement electrodes situated on both their inner and outer surfaces, forming an oxygen concentration cell. The potential reaction of this cell is directly correlated to the concentration of oxygen content present in the flue gas. Additionally, the potential obtained adheres to the Nernst equation as follows

(1) $$E=\left(\frac{RT}{zF}\right)\ln\left(\frac{P_{0}}{P}\right)$$

where E represents the potential signal of zirconia, R refers to the ideal gas constant, T represents the absolute temperature, z represents the number of electron transfers in the reaction, F denotes Faraday’s constant, $P_{0}$ signifies the oxygen content in the air, and P represents the oxygen content in the flue gas.

Zirconia cells demonstrate various local potentials during the process of measurement, encompassing the temperature potential, the contact potential, the reference potential, and polarization potential. These potentials can be restated as follows

(2) $$E_{m}=\left(\frac{RT}{4F}\right)\ln\left(\frac{P_{0}}{P}\right)+E_{0}$$

where $E_{m}$ indicates the potential signal of zirconia, and $E_{0}$ signifies the local potential.

The complex structure and high manufacturing cost of zirconia sensors make them less favorable. Moreover, during long-term online monitoring, the dust generated by the boiler’s production process may block the sensor’s probe, while corrosive substances in the flue gas can damage the battery. These factors contribute to the aging of the zirconia battery, which lead to delayed display and an increase in the local battery’s potential $E_{0}$, thereby increasing the internal resistance and inaccurate measurements. Therefore, this study used the soft measurement method to achieve real-time and accurate measurement of the oxygen content in the flue gas, replacing zirconia oxygen sensors. The formula based on the combustion principle is as follows [38]

(3) $$O_{2}=(A_{r}-A_{i}\cdot C_{r})\cdot 21/[A_{r}+(V_{i}-A_{i})\cdot C_{r}]$$

where $A_{r}$ is the total rate of air flow supplied to the furnace, $C_{r}$ represents the total coal input to the furnace, $A_{i}$ denotes the ideal air flow rate, and $V_{i}$ represents the ideal quantity of flue gas generated by the complete combustion of 1 kg of coal.

According to Formula (3), it can be observed that the oxygen content in flue gas is mainly influenced by the air flow rate of the induced draft, the primary air flow rate, and the coal flow rate. Moreover, considering the actual process, 19 parameters were preliminarily selected as auxiliary variables. These parameters included the actual load of the boiler, the main steam temperature, the main steam flow rate, etc., as shown in Table 1. The data used in this study were obtained from the historical database of the ultra-supercritical direct-fired coal-fired unit of 350 MW in a specific power plant, covering a period of about 3 months. The data were collected every hour, with a total of 2162 datasets. 

## 3. Theoretical Foundations

### 3.1. LSTM Model of the Oxygen Content in the Flue Gas 

**Figure 2:**
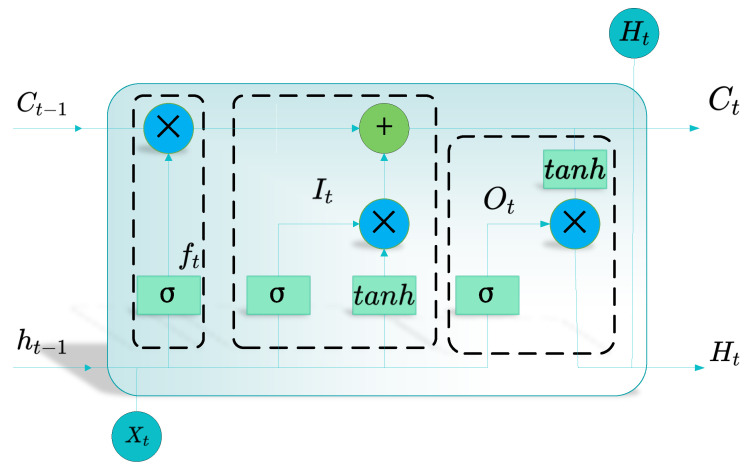
Architecture of the LSTM.

LSTM is a variant of the RNN proposed by Sepp Hochreiter and Jurgen Schmidhuber in 1997. It was primarily designed to address the issues of gradient vanishing and gradient explosion in traditional RNNs dealing with long-term dependencies [39]. Figure 2 depicts the LSTM’s architecture, which consists of four inputs and one output, along with three control gates. These three gates utilize sigmoid functions, which have values ranging from 0 to 1, to indicate the extent of openness of the gate. The activation function uses the tanh function, which has values ranging from −1 to 1, to normalize and avoid unnecessary impacts of weight caused by feature disparities. Firstly, multiple storage units within the memory block and the input gate update the cell’s state. Secondly, the forget gate determines which information to discard or retain. Finally, the output gate decides the value of the next state. By utilizing these three gates, LSTM effectively controls the historical information, gathers the external data, and filters the internal data.

- Forget gate

(4) $$f_{t}=\sigma \left(W_{f}\cdot \left[h_{t-1},x_{t}\right]+b_{f}\right)$$

- Input gate

(5) $$i_{t}=\sigma \left(W_{i}\cdot \left[h_{t-1},x_{t}\right]+b_{i}\right)$$

(6) $${\tilde{C}}_{t}=\tanh\left(W_{C}\cdot \left[h_{t-1},x_{t}\right]+b_{c}\right)$$

- State of the cell

(7) $$C_{t}=f_{t}\times C_{t-1}+i_{t}\times {\tilde{C}}_{t}$$

- Output gate

(8) $$o_{t}=\sigma \left(W_{o}\left[h_{t-1},x_{t}\right]+b_{o}\right)$$

(9) $$h_{t}=o_{t}\times \tanh\left(c_{t}\right)$$

The formula includes the variables $\;W_{f}$, $W_{i}$, $W_{c}$, and $W_{o}$, which represent the weights of the forget gate, the input gate, the output gate, and the input unit states. The variables $b_{f},{\;b}_{i},b_{c}$, and $b_{o}$ are the bias vectors; σ denotes the sigmoid function. The variables $c_{t}$ and $c_{t-1}$ represent the memory at times *t* and *t* − 1. Similarly, the variables $h_{t}$ and $h_{t-1}$ represent the output states at times *t* and *t* − 1. The variable ${\tilde{C}}_{t}$ represents the input state at times *t*, and $x_{t}$ represents the input at time *t*.

### 3.2. The Whale Optimization Algorithm

The WOA, serving as a novel heuristic optimization algorithm, mimics the hunting behavior of humpback whales and belongs to the category of swarm intelligence algorithms. Similar to PSO, the algorithm primarily comprises three operators: encircling the prey and using bubble-net attack methods to achieve the local search, and a random search strategy to implement the global search.

(1) Encircling prey

Prior to the search, it is crucial to identify and encompass the position of the prey. Since the optimal position in the search space is unknown, the WOA assumes that the current best candidate solution is either the prey or close to optimal. In the WOA, the assumption is made that the remaining search agents will update their positions in an effort to approach the most optimal agent. The calculation formula is as follows

(10) $$D=\left|C\cdot X^{*}\left(t\right)-X\left(t\right)\right|$$

(11) $$X\left(t+1\right)=X^{*}\left(t\right)-A\cdot D$$

where *t* represents the current iteration count, *A* and *C* are coefficient vectors, $X^{*}(t)$ denotes the current position of the optimal solution, and *X*(*t*) signifies the position vector of a whale. The method of calculation for the vectors *A* and *C* is as follows:

(12) $$A=2aXr_{1}-a$$

(13) $$C=2\times r_{2}$$

Throughout the entire iteration process, a linearly decreases from 2 to 0. The vectors $r_{1}$ and $r_{2}$ are random vectors in the range of [0, 1].

(2) Bubble-net attack method

**Figure 3:**
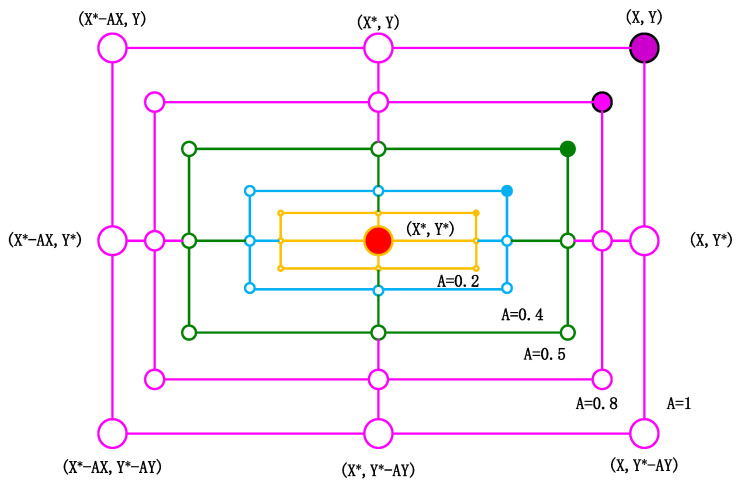
Shrink-wrap mechanism.

When engaged in hunting, whales mainly use two hunting methods: encircling prey and bubble-net attacks. Figure 3 illustrates the essence of predation in a 2D space. When *A*∈ [0, 1], it displays all *(X*, *Y*) positions that may exist between the original position of the agent and the highest position of the agent.

A bubble-net attack entails the whale and its prey constantly adjusting their positions, which can be represented through the logarithmic spiral equation, as expressed in the following equation

(14) $$X\left(t+1\right)=D^{\prime }\times e^{bl}\times \cos\left(2\pi l\right)+X^{*}\left(t\right)$$

(15) $$D^{\prime }=\left|X^{*}\left(t\right)-X\left(t\right)\right|$$

where $D^{\prime }$ represents the distance between an individual search and the current optimal solution, *b* represents a constant for the spiral shape, and *l* is a random number uniformly distributed within the interval [−1, 1].

For improved coordination between the two phases of contraction enclosure and updating the spiral, this method utilizes the probability *p* to determine the phase of updating the whale’s position; *p* ranges from 0 to 1, and its mathematical model is illustrated below:

(16) $$X\left(t+1\right)=\left\{\begin{array}{c} X_{p}\left(t\right)-A\cdot D\;\;\;\;\;\;\;\;\;\;\;\;\;\;\;\;\;\;\;\;\;\;\;\;\;\;\;\;p<0.5\\ D\cdot e^{bl}\cdot \cos\left(2\pi l\right)+X_{p}\left(t\right)\;\;\;\;\;\;\;\;\;p\geq 0.5 \end{array}\right.$$

(3) Searching for prey

The value of |*A*| can determine whether the whale is in either the food-searching phase, or the surrounding and hunting phase. The WOA updates the position based on the distances between the whales to achieve random searching. Therefore, when |*A*| ≥ 1, it indicates that the whale has not obtained useful information. As a result, it will enter the food-searching phase. The search model is established based on the random walk method during searching for food, as shown below

(17) $$D^{\prime \prime }=\left|C\cdot X_{rand}\left(t\right)-X\left(t\right)\right|$$

(18) $$X\left(t+1\right)=X_{rand}\left(t\right)-A\times D$$

where $D^{\prime \prime }$ represents the distance between the previous individual search and the random individual. $X_{rand}\left(t\right)$ represents the position of the previous random individual.

**Figure 4:**
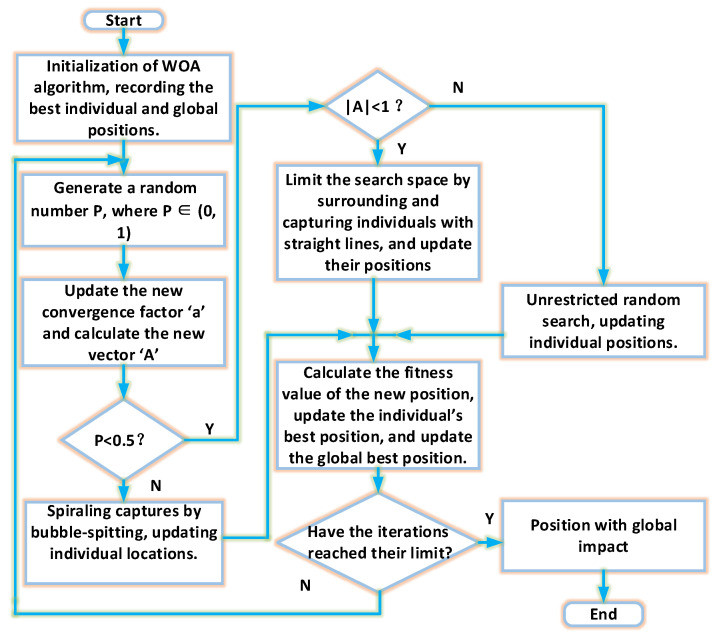
The flowchart of the WOA.

Figure 4 displays the flowchart for the WOA.

### 3.3. WOA-LSTM

**Figure 5:**
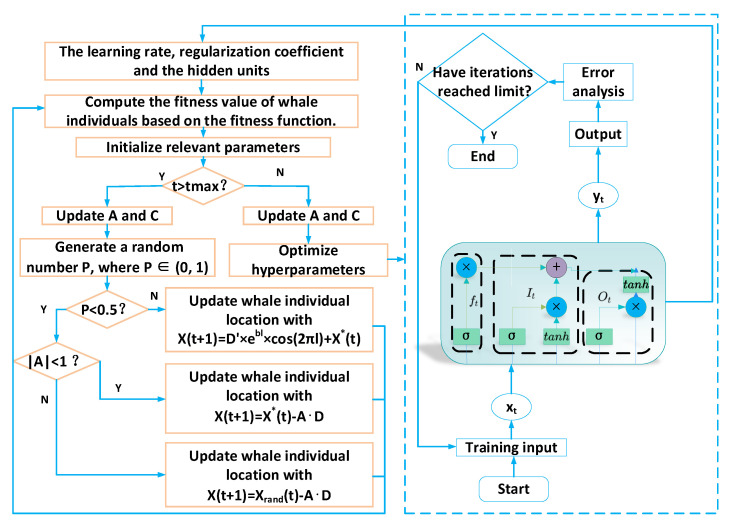
The flowchart of the WOA-LSTM.

Many studies have illustrated the importance of the selection of hyperparameters in LSTM models. However, the LSTM model requires manual setting of the learning rate, the regularization coefficient, and the hidden units, and it is not an easy task to find the optimal parameter settings that enable the neural network to achieve the best measurement accuracy within a certain range. To address this issue, the WOA was introduced to optimize the parameters of LSTM neural network, and the optimal learning rate, the number of hidden layer neurons, and the regularization coefficient could be determined. Figure 5 illustrates the process of predicting the oxygen content using the WOA-LSTM.

The algorithm’s process is as follows:

(a) The relevant parameters of the LSTM are initialized, which include the learning rate, the regularization coefficient, and the hidden units.
(b) The parameters of the whale optimization algorithm are initialized, which include the maximum number of iterations *t_max_*, the number of whales *n*, the upper limit of the search range *ub*, and the lower limit *lb*.
(c) Compute the fitness of each whale, identify the current optimal whale’s position, and retain it.
(d) Update the coefficient vectors *A* and *C*. If the probability *P* is less than 50%, proceed to the next step; if not, use the mechanism of bubble-net feeding.
(e) If the absolute value of the coefficient vector *A* is smaller than 1, surround the prey; otherwise, the prey is searched for globally and randomly.
(f) The WOA continuously optimizes the network’s parameters until the iterations end, obtaining the optimal learning rate, the number of neurons in the hidden layer of the neural network, and the regularization coefficient.
(g) Based on the best combination of parameters, output the soft sensing value of the oxygen content in the flue gas.

## 4. Influencing Attributes of the Output of the Oxygen Content of Flue Gas 

### 4.1. Pearson’s Correlation

Pearson’s correlation is a statistical method used to quantify the correlation between two variables. In coal-fired power plants, Pearson correlation analysis was used to explore the relationship between the oxygen content of flue gas and other parameters. 

Given two *n*-dimensional vectors *X* = [$X_{1}$, $X_{2}$, *…*, $X_{n}$] and [$Y_{1}$, $Y_{2}$,…, $Y_{n}$], the correlation coefficient between them can be calculated as follows

(19) $$\rho_{\left(X,Y\right)}=\frac{\mathrm{cov}\left(X,Y\right)}{\sigma_{X}\sigma_{Y}}=\frac{\sum_{i=1}^{n}\left(X_{i}-\bar{X}\right)\left(Y_{i}-\bar{Y}\right)}{\sqrt{\sum_{i=1}^{n}(X_{i}-\bar{X}{)}^{2}\sum_{i=1}^{n}(Y_{i}-\bar{Y}{)}^{2}}}$$

where $\bar{X}$ and $\bar{Y}\;$ represent the mean values of the variables *X* and *Y*.

**Figure 6:**
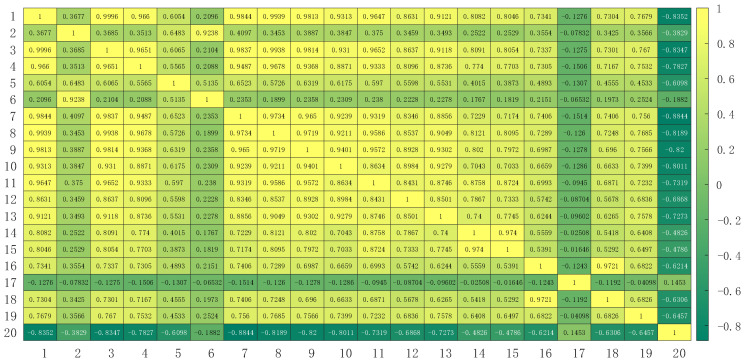
Heatmap of Pearson’s correlation coefficient.

The heatmap of Pearson’s correlation coefficient is shown in Figure 6, which is used to display the strength of the correlations among different variables. Typically, the values of Pearson’s correlation coefficient range from −1 to 1, where 1 indicates a perfect positive correlation, −1 indicates a perfect negative correlation, and 0 indicates no correlation. The intensity of the colors reflects the magnitude of the values, shown as a brighter color in the heatmap.

According to the data presented in Table 2, there is a noteworthy relationship between each input variable and the oxygen content observed in the flue gas. Among them, eight input variables showed a stronger association with the oxygen content, and six input variables displayed an even stronger association with the oxygen content. However, there were five input variables that demonstrated a relatively weak correlation with the oxygen content. It was necessary to exclude these five variables from the modeling process. These variables comprised the flue gas pressure at the low-temperature reheater’s outlet, the superheater outlet header’s outlet temperature, the main steam temperature, the #1 supply fan’s current, and the #2 supply fan’s current.

### 4.2. Principal Component Analysis (PCA)

In practical engineering, numerous variables may be influenced by the same driving variable, thus leading to redundancy in the information. To address this situation, the application of PCA can be used. PCA is a statistical method for projecting high-dimensional data into a low-dimensional subspace. The purpose is to reduce the dimensions of the data while preserving as much of the process’s information as possible, which helps to the extract key features and simplifies the complexity of the dataset, enhancing the efficiency and improving the accuracy of the model [40].

PCA was performed on sample data from coal-fired power plants to obtain the principal components after reductions in the dimensionality [41]. The steps of the PCA are described in detail as follows.

(1) Structure the entire historical dataset of the load into a sample matrix with the dimensions *m* × *n*
  (20) $$\left.\left[\begin{array}{ccc} a_{11} & ... & a_{1n}\\ a_{21} & ... & a_{2n}\\ ... & ... & ...\\ a_{m1} & ... & a_{mn} \end{array}\right.\right]$$
(2) Compute the mean value of the *n*-dimensional dataset *A*, where $A=\left\{a_{1},a_{2},\ldots ,a_{m}\right\}$
  (21) $$\overset{-}{A}=\frac{1}{m}\sum_{i=1}^{m}A_{i}$$
  where *m* is the total number of samples, and $\overset{-}{A}$ is the obtained sample mean.
(3) Calculate the covariance matrix of the sample set using the generated sample mean.
  (22) $$Cov(A,B)=\frac{\sum_{i=1}^{m}\left(A_{i}-\overset{-}{A}\right)\left(B_{i}-\overset{-}{B}\right)}{m}$$
  where Cov(A,B) is the covariance matrix of the sample set.
(4) Calculate the eigenvectors and eigenvalues of the covariance matrix
  (23) $$Cov(A,B)=Q\cdot \sum \cdot Q^{T}$$
  (24) $$\sum =diag(\lambda_{1},\lambda_{2},\ldots ,\lambda_{n})\lambda_{1}\geq \lambda_{2}\geq \cdots \geq \lambda_{n}\geq 0$$
  (25) $$Q=[q_{1},q_{2},\ldots ,q_{n}]$$
  where ∑ is the diagonal matrix of *n* eigenvalues arranged in descending order in the covariance matrix, $\lambda_{i}$ is the eigenvalue corresponding to the covariance matrix, and the feature matrix *Q* is composed of the feature vectors $q_{i}$ corresponding to the eigenvalues $\lambda_{i}$.
(5) Calculate the cumulative contribution to variance of the top *k* principal components by using the obtained eigenvalues and eigenvectors
  (26) $$\vartheta =\frac{\lambda_{1}+\lambda_{2}+...+\lambda_{k}}{\lambda_{1}+\lambda_{2}+...+\lambda_{p}}$$
  where $\lambda_{k}$ is *k*-th eigenvalue, and *p* presents the number of variables.
(6) By projecting the original data into a *k*-dimensional subspace and selecting the top *k* eigenvectors of the covariance matrix, a new basis for the data is constructed.
  (27) $$a_{i}^{new}=\left[\begin{array}{c} Q_{1}a_{k}\\ Q_{2}a_{k}\\ ......\\ ......\\ Q_{k}a_{k} \end{array}\right]\in A_{k}$$

By using the method above, the original data were linearly transformed from *n* to *k* dimensions to achieve the goal of reducing the dimensionality.

**Figure 7:**
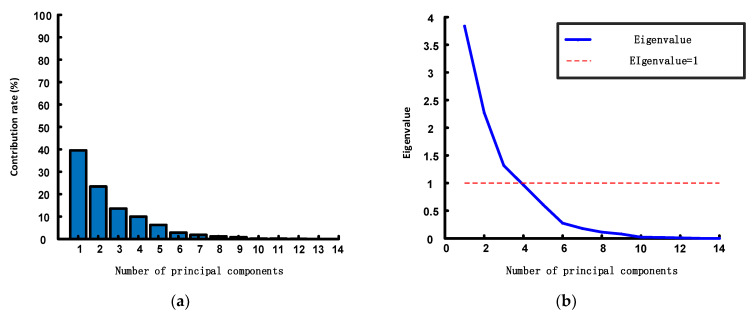
Principal component analysis (PCA). (**a**) Contribution rate; (**b**) eigenvalues.

**Figure 8:**
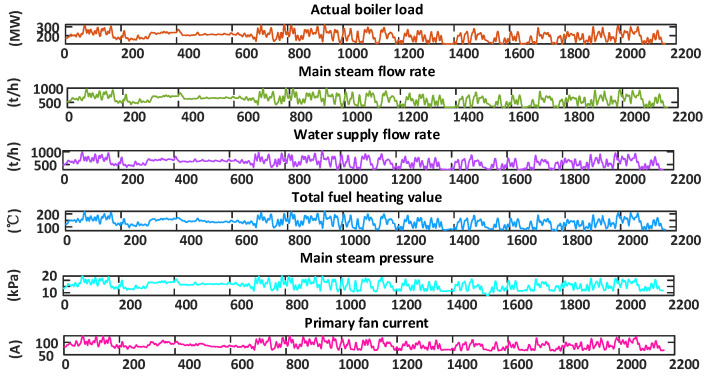
Auxiliary variables.

In PCA, eigenvalues serve as indicators to assess the relationships among variables within a dataset. The larger the eigenvalue, the greater the variance explained by the corresponding principal component, indicating the greater contribution of this principal component to the dataset. Generally, when the cumulative contribution rate falls within the range of 80% to 95%, the first *k* principal components effectively capture most of the original data’s information. For this study, a 95% cumulative contribution rate was required. As depicted in Figure 7, the cumulative proportion yielded by the initial six principal components surpassed 95%. Therefore, after variable selection based on PCA and a reduction in the dimensionality, six principal components were ultimately selected as the auxiliary variables. Figure 8 shows the final auxiliary variables determined by this process.

### 4.3. Preprocessing of Data

Appropriate preprocessing of the historical data from the power plant should be performed prior to establishing a soft measurement model. 

(1) The 3*σ* criterion is commonly used for identifying abnormal data points within samples, as defined in Equation (28).

(28) $$V_{p}=X_{p}-\overset{-}{X}$$

where $V_{p}$ (*p* = 1, 2, …, *n*) represents the residual error corresponding to the *p*th data point in the sample.

Calculate the standard deviation *σ* of the entire sample:

(29) $$\sigma =\sqrt{\frac{1}{n}\sum_{p=1}^{n}{\left(X_{p}-\overset{-}{X}\right)}^{2}}$$

After the absolute value of the residual error for each data point has been calculated, it is sequentially compared with 3*σ*. If it satisfies Equation (30), data point $X_{p}$ is classified as an outlier.

(30) $$|V_{p}|>3\sigma$$

(2) The five-point cubic smoothing algorithm was used in this study to handle random errors and to remove random disturbances. The five-point cubic smoothing algorithm belongs to the category of smoothing filter algorithms which utilize polynomial least squares approximation for the approximation of sampling points and achieving smooth filtering. The formula for the five-point cubic smoothing is presented below:

(31) $${\overset{-}{Y}}_{-2}=\frac{69Y_{-2}+4Y_{-1}-6Y_{0}+4Y_{1}-Y_{2}}{70}$$

(32) $${\overset{-}{Y}}_{-1}=\frac{2Y_{-2}+27Y_{-1}+12Y_{0}-8Y_{1}+2Y_{2}}{35}$$

(33) $$\left.{\overset{-}{Y}}_{0}=\right.\frac{\left(\begin{array}{c} -3Y_{-2}+12Y_{-1}+17Y_{0}+12Y_{1}-3Y_{2}) \end{array}\right.}{35}$$

(34) $$\left.{\overset{-}{Y}}_{1}=\right.\frac{\left(\begin{array}{c} 2Y_{-2}-8Y_{-1}+12Y_{0}+27Y_{1}+2Y_{2}) \end{array}\right.}{35}$$

(35) $${\overset{-}{Y}}_{2}=\frac{-Y_{-2}+4Y_{-1}-6Y_{0}+4Y_{1}+69Y_{2}}{70}$$

## 5. Simulation Results

### 5.1. Evaluation Indicators

The choice of a soft measurement model for the oxygen content of flue gas should adhere to various performance metrics. The root mean square error (*RMSE*), mean square error (*MSE*), mean absolute percentage error (*MAPE*), mean absolute error (*MAE*), and R-squared ($R^{2}$) were used to evaluate the performance of the predictive models.

(36) $$MAE=\frac{\Sigma_{i}^{n}\left|{\hat{y}}_{i}-y_{i}\right|}{n}$$

(37) $$RMSE=\sqrt{\frac{\sum_{i}^{n}(\hat{y_{i}}-y_{i}{)}^{2}}{n}}$$

(38) $$MAPE=\frac{\sum_{i}^{n}\left|\hat{y_{i}}-y_{i}\right|}{y_{i}}\times \frac{100\%}{n}$$

(39) $$R^{2}=1-\frac{\Sigma_{i}^{n}(\hat{y_{i}}-y_{i}{)}^{2}}{\Sigma_{i}^{n}(\overset{-}{y_{i}}-y_{i}{)}^{2}}$$

In the equations above, i = 1, 2, 3, …, n, where n represents the number of experiments; $\hat{y_{i}}$ represents the predicted value; and $y_{i}$ denotes the true value.

### 5.2. Experimental Results

This study collected historical data from coal-fired power plants from January to April in 2023, with a time interval of 1 hour. The training sets were assigned 80% of the data, and the testing sets were assigned the remaining 20%. In this simulation, the hardware parameters were as follows: the CPU used was the Intel^®^ Core™ i7-7300HQ (Intel, Santa Clara, CA, USA), running at a clock frequency of 2.50 GHz, with a memory capacity of 8 GB. The deep learning model was constructed using MATLAB2022a. In order to assess the accuracy and effectiveness of the proposed model, this study conducted a comprehensive comparison of various soft measurement models. The parameters’ values are shown in Table 3.

**Figure 9:**
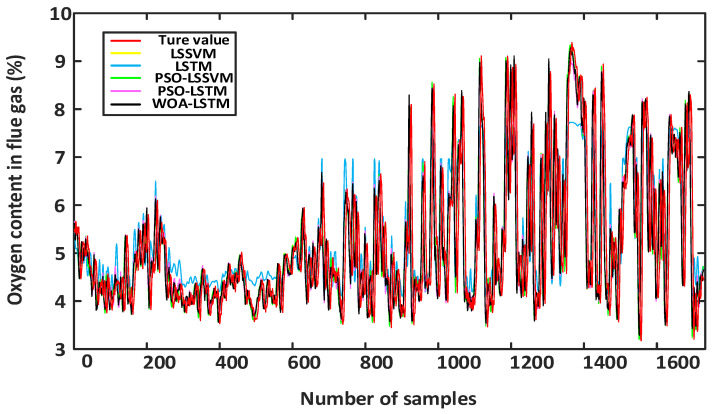
Training results of different models.

Figure 9 shows a comparison of five models with the training sets. It was observed that the performance of the single LSTM model was poor in the training phase, while the performance of the LSSVM model surpassed that of the hybrid model on the training sets. However, during the prediction phase, the performance of the single LSSVM model in predicting the oxygen content in flue gas from coal-fired power plants was unsatisfactory, deviating significantly from the actual measured values. This could be due to overfitting of the LSSVM model during the training phase, resulting in poor performance on the test sets. Moreover, when there is a significant difference in the feature distribution between the training sets and test sets, the model may overly rely on the feature distribution of the training sets, resulting in poor performance on the test sets. Although the WOA-LSTM model did not perform as well as the LSSVM model on the training sets, it performed the best in predicting the oxygen content in the flue gas on the test sets, demonstrating good adaptability.

**Figure 10:**
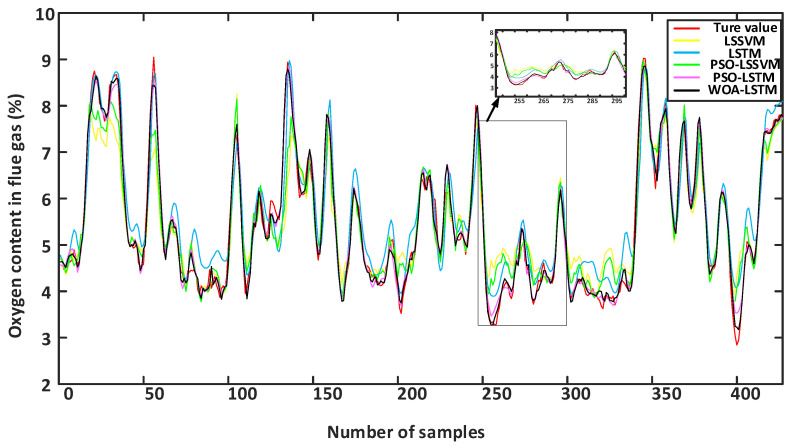
Predicted results of different models.

Figure 10 presents a comparison among the five models, indicating that the performance of the single LSSVM model was unsatisfactory in predicting the oxygen content in the flue gas from coal-fired power plants, with a significant deviation from the actual measured values. This was due to the weaker ability of LSSVM models to extract the data’s features and their lower stability when dealing with longer dynamic sequences. Although the LSTM model exhibited higher prediction accuracy compared with the LSSVM model, there was still a significant difference between the predicted oxygen content in the flue gas and the actual oxygen content, which did not fulfil the demands for predicting the oxygen content in the flue gas.

To enhance the precision of soft measurement, this study used the PSO optimization method to determine the parameters of LSSVM [42]. In addition, in order to optimize the hyperparameters in LSTM, this study used the PSO and WOA methods for parameter optimization. Table 4 presents the optimization results of the same hyperparameters in LSTM using both the PSO and WOA optimization algorithms. From Figure 8, it can be seen that the PSO-LSSVM model demonstrated improvements in the predictive accuracy to a certain degree, while the PSO-LSTM model performs better in terms of the accuracy of soft measurement. In contrast, the WOA-LSTM model performed best in predicting the oxygen content in flue gas and had good adaptability. Therefore, the WOA method can automatically adjust the search strategy based on the characteristics of the problem, improve the adaptability of the model, and provide more accurate prediction results. 

### 5.3. Experimental Analysis and Comparison

**Figure 11:**
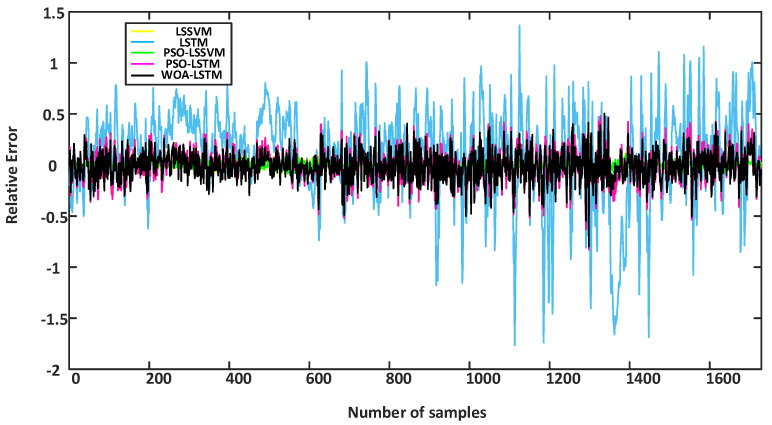
Training results for the relative errors among different models.

**Figure 12:**
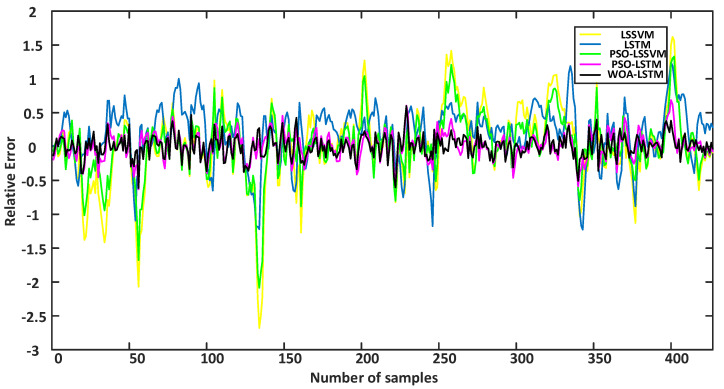
Test results for the relative errors among different models.

To effectively assess diverse models, this article presents the error curves of each model for the training sets and testing sets in Figure 11 and Figure 12. Additionally, the corresponding error data are provided in tabular form, as shown in Table 5.

According to the results shown in Figure 11, during the training phase, the relative error fluctuated significantly for the single LSTM model. This indicates that the single LSTM model exhibited fluctuations in the training process, whereas the other models showed smoother performance in terms of the relative error. Figure 12 shows that the WOA-LSTM model performed better in terms of relative error compared with other methods. The error metrics are displayed in Table 5, which indicate that the WOA-LSTM model achieved an improvement in accuracy of 0.49% compared with the PSO-LSTM model, 1.35% compared with the PSO-LSSVM model, 4.03% compared with the stand-alone LSTM model, and 4.93% compared with the LSSVM model. The WOA used an “attack” strategy during the search process by randomly selecting individuals to enhance the search for the global optimal solution. 

Additionally, *R*^2^ was statistically analyzed within this study. The findings revealed that the WOA-LSTM achieved the highest value for *R*^2^ compared with other prediction models, indicating improvements in the explanatory ability and degree of fitting of the prediction model. It is evident from the graphs of the prediction results, the error graphs, and the tables that the soft sensor model proposed in this study surpassed other prediction models.

**Figure 13:**
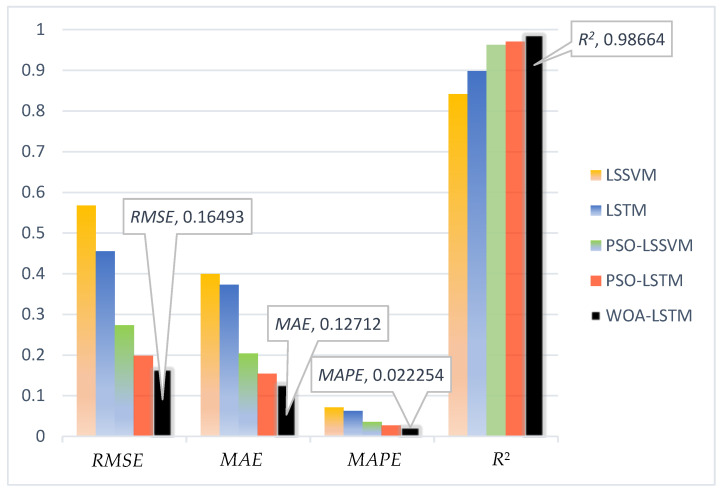
Comparative analysis of the error of predicted oxygen content for the testing sets using various prediction models.

To enhance our understanding of the forecast outcomes, this study used a visually appealing histogram, and Figure 13 illustrates the results of the comparison. For *RMSE* and *MAE*, the *y*-axis represents the measure of the error values, with smaller values indicating the stronger predictive ability of the model for the oxygen content in flue gas. For *R*^2^, the value of the *y*-axis represents the goodness of fit of the model to the data on the oxygen content of flue gas, with values ranging from 0 to 1. When *R*^2^ is close to 1, this indicates that the model can adequately explain the variation in the data on the oxygen content of flue gas; when *R*^2^ is close to 0, it means that the model’s fit is poor. For *MAPE*, the values on the *y*-axis represent the mean absolute percentage of error between the predicted values and the true values, expressed as a percentage. In these visuals, the color orange signifies the LSSVM model, blue represents the LSTM model, green denotes the PSO-LSSVM model, red symbolizes the PSO-LSTM model, and black denotes the WOA-LSTM model. Notably, from the graphical representation, it is evident that the bar corresponding to the WOA-LSTM is the shortest, suggesting minimal predictive errors and the high precision of this proposed model.

## 6. Conclusions

The combustion system in coal-fired power plant boilers is a complex nonlinear system with a time delay, time characteristics, and interdependence between various factors. Historical data collected from coal-fired power plants often contain noise and outliers. Therefore, this study used the method of using 3*σ* and smoothing curves to handle abnormal data. In order to address the challenge of dealing with the extensive and abundant data found in coal-fired power plants, this study utilized PCA to extract the significant features for predicting the oxygen content in flue gas. Additionally, the article introduces a combined WOA-LSTM model to enhance the precision of predicting the oxygen content in the flue gas. By comparing it with other models and analyzing it, the results indicated that the hybrid WOA-LSTM model exhibited high accuracy and applicability in precisely predicting the oxygen content in the boiler flue gas. In our future research, we will endeavor to achieve real-time soft measuring of the gas oxygen content in a 350 MW power generation facility, thereby further validating the efficacy of the proposed method.

## Figures and Tables

**Table 1:** Selected variables in the system.

No.	Input Variables	Maximum Value	Minimum Value	Unit
1	Actual boiler load	353	101.12	MW
2	Main steam temperature	571.9	517.13	°C
3	Main steam flow rate	1079.76	307.08	t/h
4	Main steam pressure	24.75	7.64	MPa
5	Outlet steam temperature of reheater	568.39	491.15	°C
6	Superheater outlet header’s outlet temperature	574.66	498.43	°C
7	Water temperature of boiler feed	280	212.67	°C
8	Water supply flow rate	115.75	282.79	t/h
9	Total fuel heating value	245.27	67.4	kJ/°C
10	Total primary air volume	443.54	204.77	t/h
11	Total secondary air volume	810.76	247.44	t/h
12	#1 Primary fan current	142.43	65.57	A
13	#2 Primary fan current	169.95	67.23	A
14	#1 Supply fan current	46.6	0.14	A
15	#2 Supply fan current	49.91	15.8	A
16	Flue gas temperature of low-temperature superheater	589.62	412.85	°C
17	Outlet flue gas pressure of low temperature reheater	−0.04	−0.49	kPa
18	Temperature of flue gas at the outlet of the economizer	440.97	300.56	°C
19	Flue gas temperature of air preheater outlet	144.76	103.36	°C

**Table 2:** Pearson’s correlation coefficients.

Input Variables	Correlation
Boiler feed	0.8844
Actual boiler load	0.8352
Main steam flow rate	0.8347
Total fuel heating value	0.82
Flow rate of the water supply	0.8189
Total primary air volume	0.8011
Main steam pressure	0.7827
Total secondary air volume	0.7319
#2 Primary fan current	0.7273
#1 Primary fan current	0.6868
Flue gas temperature at the air preheater outlet	0.6457
Temperature of flue gas at the outlet of the economizer	0.6306
Flue gas temperature of the low-temperature superheater	0.6214
Outlet steam temperature of reheater	0.6098
#1 Supply fan current	0.4826
#2 Supply fan current	0.4786
Main steam temperature	0.3829
Superheater outlet header’s outlet temperature	0.1882
Flue gas pressure at the low-temperature reheater outlet	0.1453

**Table 3:** Parameters for the LSTM model.

Parameter	Numerical Value
Delay time step	5
Population size	10
Learning rate	[0.001, 0.01]
Maximum iterations	100
Count of the hidden layers’ nodes	[16, 72]
Regularization parameter	($1\times 10^{-6},1\times 10^{-5}$)

**Table 4:** The ultimate selection of hyperparameters.

Model	Learning Rate	Regularization Coefficient	Hidden Units
WOA-LSTM	0.0083	$9.1441\times 10^{-6}$	43
PSO-LSTM	0.0073	$7.077\times 10^{-6}$	20

**Table 5:** Comparison of the errors for five models.

	Model	*RMSE*	*MAE*	*MAPE*	$R^{2}$
Training set	LSSVM	0.19375	0.13189	0.25188%	0.99979
	LSTM	0.4494	0.34872	6.8337%	0.92652
	PSO-LSSVM	0.04384	0.03164	0.58173%	0.99891
	PSO-LSTM	0.17671	0.13837	2.6316%	0.98231
	WOA-LSTM	0.14019	0.10789	2.0025%	0.98864
Test set	LSSVM	0.56778	0.39939	7.1514%	0.84169
	LSTM	0.45506	0.3729	6.255%	0.89831
	PSO-LSSVM	0.27478	0.20416	3.5814%	0.96292
	PSO-LSTM	0.19836	0.15445	2.7191%	0.97068
	WOA-LSTM	0.16493	0.12712	2.2254%	0.98664

## Data Availability

The data presented in this study are available on request from the corresponding author. The data are not publicly available due to privacy.
